# Evaluation of the stability of the stereotactic Leksell Frame G in Gamma Knife radiosurgery

**DOI:** 10.1120/jacmp.v17i3.5944

**Published:** 2016-05-08

**Authors:** Alvaro Rojas‐Villabona, Katherine Miszkiel, Neil Kitchen, Rolf Jäger, Ian Paddick

**Affiliations:** ^1^ Department of Neurosurgery National Hospital for Neurology and Neurosurgery Queen Square London UK; ^2^ The Gamma Knife Centre at Queen Square National Hospital for Neurology and Neurosurgery Queen Square London UK; ^3^ The Lysholm Department of Neuroradiology National Hospital for Neurology and Neurosurgery Queen Square London UK

**Keywords:** radiosurgery, stereotactic techniques, quality assurance

## Abstract

The purpose of this study was to evaluate the stability of the Leksell Frame G in Gamma Knife radiosurgery (GKR). Forty patients undergoing GKR underwent pretreatment stereotactic MRI for GKR planning and stereotactic CT immediately after GKR. The stereotactic coordinates of four anatomical landmarks (cochlear apertures and the summits of the anterior post of the superior semicircular canals, bilaterally) were measured by two evaluators on two separate occasions in the pretreatment MRI and post‐treatment CT scans and the absolute distance between the observations is reported. The measurement method was validated with an independent group of patients who underwent both stereotactic MRI and CT imaging before treatment (negative controls; n: 5). Patients undergoing GKR for arteriovenous malformations (AVM) also underwent digital subtraction angiography (DSA), which could result in extra stresses on the frame. The distance between landmark localization in the scans for the negative control group (0.63 mm; 95% CI: 0.57–0.70; SD: 0.29) represents the overall consistency of the evaluation method and provides an estimate of the minimum displacement that could be detected by the study. Two patients in the study group had the fiducial indicator box accidentally misplaced at post‐treatment CT scanning. This simulated the scenario of a frame displacement, and these cases were used as positive controls to demonstrate that the evaluation method is capable of detecting a discrepancy between the MRI and CT scans, if there was one. The mean distance between the location of the landmarks in the pretreatment MRI and post‐treatment CT scans for the study group was 0.71 mm (95% CI: 0.68–0.74; SD:0.32), which was not statistically different from the overall uncertainty of the evaluation method observed in the negative control group (p=0.06). The subgroup of patients with AVM (n: 9), who also underwent DSA, showed a statistically significant difference between the location of the landmarks compared to subjects with no additional imaging: 0.78 mm (95% CI: 0.72–0.84) vs. 0.69 mm (95% CI: 0.66–0.72), p=0.016. This is however a minimal difference (0.1 mm) and the mean difference in landmark location for each AVM patient remained submillimeter. This study demonstrates submillimeter stability of the Leksell Frame G in GKR throughout the treatment procedure.

PACS number(s): 87.53.‐j, 87.53.Ly, 87.56.Fc

## I. INTRODUCTION

Gamma Knife radiosurgery (GKR) has traditionally relied on a rigid immobilization system to stereotactically converge multiple beams of ionizing radiation at a defined intracranial target. The procedure is aimed to eradicate or inactivate the target, and the biological effect of the energy delivered is expected to affect all the structures within the volume of the prescribed dose, while minimizing exposure to the surrounding tissue.^(1)^ Therefore, the accuracy of the stereotactic system, which encompasses localization of the targets with minimal spatial error and a high degree of reproducibility, is of paramount importance for safe delivery of GKR.^(2)^

The overall application accuracy of GKR, also referred to as the total clinically relevant error,^(2)^ is the result of individual inaccuracies associated with each step in the procedure and they can be grouped into three categories. First is the difference between the radiation delivered to the patient and that defined in the treatment plan in terms of magnitude, location or distribution. This can result from errors or approximations in dose calculation or due to mechanical inaccuracy of the treatment delivery itself, which is the estimate of accuracy usually provided by the manufacturers. The second category refers to inaccurate definition of the target which results from erroneous imaging technique selection, inappropriate interpretation of the images, or geometrical inaccuracies of the scans due to distortion or other technical factors.^(3)^ This latter error has been well described and is usually assessed in standard QA procedures.^(4)^ The third source of error, which is the main scope of this study, is the possibility that the actual location of a target within the stereotactic system at treatment differs from its calculated location due to displacement of the reference frame between the imaging procedure and the treatment. Such displacement could occur from instability due to inadequate frame placement or from stresses induced in the frame between imaging and treatment. This may be more likely in the case of multiple image studies (e.g., MRI, CT, and DSA). This source of error tends to be overlooked by studies evaluating the accuracy of stereotactic systems by assuming that the frame is rigid and therefore stable, without actively testing it for potential displacements. Also, most studies investigating the stability of fixation systems are laboratory‐ and phantom‐based and standard QA procedures are not capable of detecting potential frame displacement throughout the clinical procedure.^(5)^

The most commonly used immobilization and localization method for GKR is the Leksell stereotactic coordinate frame (Elekta AB, Stockholm, Sweden), which is a dedicated stereotactic radiosurgery tool introduced by the Swedish Neurosurgeon Lars Leksell in the 1970s and further developed over the last few decades.^(6)^ The Leksell Frame G was extensively used as a stereotactic tool for brain biopsies before neuronavigation systems were developed, and it is widely used for GKR and insertion of deep brain stimulation (DBS) electrodes.^(7)^ The later procedure involves localization of very small targets, such as the subthalamic nucleus and ventral intermediate nucleus of the thalamus, and the level of precision required for such task is comparable to the accuracy required for targeting of the trigeminal nerve in GKR where a few millimeters of error could result in completely missing the target.^(8)^ This reinforces the importance of actively evaluating the stability of the Leksell frame during GKR, which is the main aim of this study.

## II. MATERIALS AND METHODS

### A. Patients

Forty consecutive patients undergoing GKR for a variety of intracranial diseases between September 2013 and June 2014 underwent stereotactic MR imaging for GKR planning and stereotactic CT imaging after GKR for research purposes (i.e., evaluation of the convolution algorithm for GKR planning). The study was approved by the research ethics committee, which is the UK equivalent to the Institutional Review Board (IRB), and written consent was received from all participants. The demographic details and diagnosis of the subjects included in the study are shown in Table 1.

**Table 1:** Demographic details and diagnosis of the study subjects.

*Age mean (min‐max)*	*53.4 yr (26–76)*	
Female, n (%)	23 (57.5%)	
Diagnosis, n (%)	Acoustic neuroma	16 (40%)
	Meningioma	10 (25%)
	AVM	9 (22.5%)
	TN	4 (10%)
	Brain metastases	1 (2.5%)
	Total	40

AVM=arterio‐venous malformation; TN=trigeminal neuralgia

### B. Stereotactic frame and pretreatment imaging

**Figure 1:**
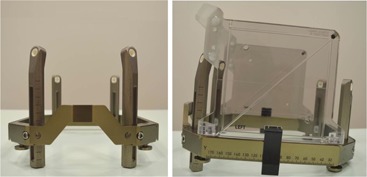
Leksell stereotactic coordinate Frame G: (left) frame assembled with the curved front piece in the upwards position, angled anterior insulated posts and medium straight insulated posts posteriorly; (right) lateral view of the Leksell Frame G with the MRI fiducial indicator box attached.

A Leksell stereotactic coordinate Frame G, assembled as shown in Fig. 1, was used for GKR. The curved front piece was in the upwards position, along with the angled anterior insulated posts (155 mm) and medium straight posts posteriorly (110 mm), which were used in most cases. Three patients had the frame applied with the front piece downwards to avoid nose compression and longer straight posts (137 mm) were used posteriorly on one patient to obtain a lower frame position. Application of the stereotactic frame was performed in a sitting position under local anesthesia and the frame was manually checked for rigidity before fitting. An injection of 5 ml of lidocaine hydrochloride 1% was given in the planned site of each of the four titanium pins, which were advanced to the outer skull table and adjusted to a consistent pin pressure of 45 cNm using a torque driver.

Stereotactic MRI for GKR planning was performed after frame application and using a Magnetom Avanto 1.5 T MRI system (Siemens AG, Erlangen, Germany). A frame MRI adapter (Elekta AB) was used to support and immobilize the patient's head and stereotactic frame on the MRI table. Postcontrast 3D T1 and T2 weighted volumetric imaging was performed: T1 weighted: Fast Low Angle SHot (FLASH); T2 weighted: Constructive Interference in Steady State (CISS); acquisition matrix: 448×448; slice thickness: 1.5 mm, no overlap; FoV: 210×210 mm; voxel size: 0.47×0.47×1.5 mm. The MRI scans were defined in stereotactic space using Leksell GammaPlan 10.1 (Elekta AB) based on fiducial markers obtained from the indicator box (Fig. 1). An appropriate radiosurgical treatment plan was developed using the aforementioned planning system and delivered using the Leksell Gamma Knife Perfexion (Elekta AB). Patients undergoing GKR for AVM (n: 9) also underwent DSA for lesion targeting and radiosurgery planning. This invasive procedure involves longer frame‐on times and increased patient handling, which could result in extra stresses on the stereotactic frame and a potentially higher risk of frame displacement.

### C. Post‐treatment imaging and landmarks measurement

A post‐GKR stereotactic noncontrast CT of the head was performed immediately after treatment and prior to frame removal using a Siemens Somatom Definition AS multislice helical CT scanner (Siemens AG, Forchheim, Germany). Acquisition matrix:512×512; slice thickness: 1.5 mm, no overlap; FoV: 240×240 mm; pixel size: 0.47×0.47 mm. Slice thickness: 1.5 mm; reconstructed FOV: 240 mm×240 mm. A frame CT adapter (Elekta AB) was used to secure the patient in the correct position, and the scans were independently defined in stereotactic space using fiducial markers from the CT indicator box.

**Figure 2:**
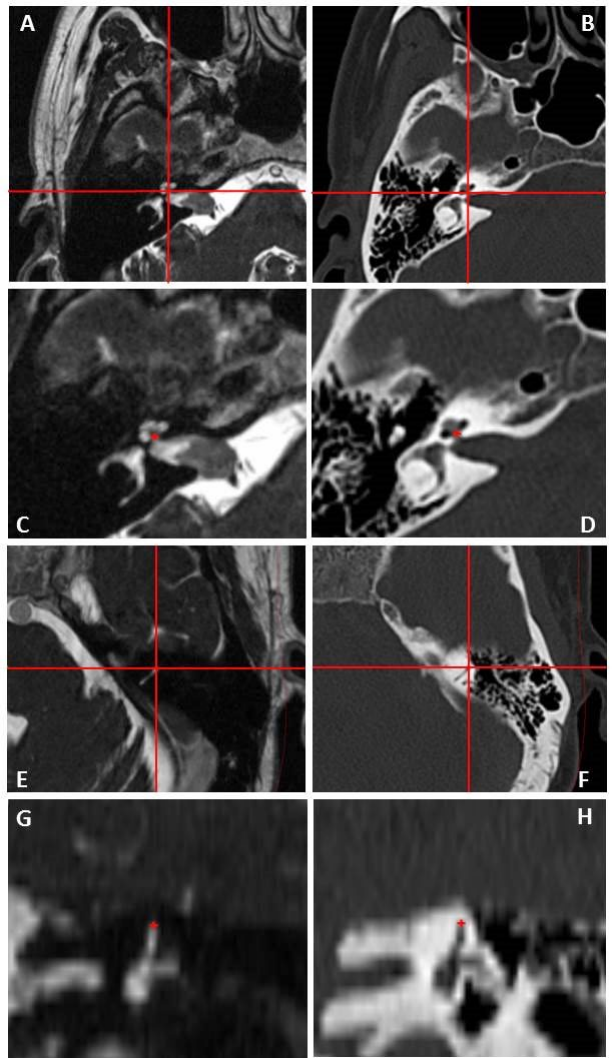
Landmarks used for frame stability evaluation. The landmarks as seen in the T2‐weighted sequence of the pre‐GKR MRI scan are showed in the left panel, while the right panel shows the same landmarks in the bone window of the post‐treatment CT scan. Right cochlear apertures ((a) and (b)) are identified by the intersection of the lines. A detail ((c) and (d)) shows the red cross on the landmark as measured for the study. Initial identification ((e) and (f)) of the anterior post of the left superior semicircular canal in the axial plane (line intersection) and its summit as identified in the coronal plane (red cross in (g) and (h)).

The stereotactic coordinates of four landmarks were measured in the pretreatment MRI and again in the post‐treatment CT scans by two different evaluators on two separate occasions with at least one week's difference between repeated measures. Figure 2 shows the landmarks used for evaluation: bilateral cochlear apertures, at the base of the modiolus, and the summits of the anterior post of the Superior Semi‐circular Canals (SSC). The landmark coordinates in the pretreatment MRI scan were taken from the T2‐weighted images and from the bone reconstruction on the post‐treatment CT scan, as shown in Fig. 2. The stereotactic coordinates of the cochlear apertures were measured in the axial plane. In two study cases, one of the cochlear apertures was bisected by two slices and this resulted in discrepancies in the Z coordinate reflecting the slice thickness rather than the apparent location of the landmark. A consensus was reached between the observers for these two cases and measurements were obtained from the same slice. To minimize the effect of slice thickness on the results, the stereotactic location of the SSC was taken from the reconstructed coronal plane. The distance between the stereotactic location of the landmarks observed in the pretreatment MRI scan and the post‐treatment CT scan was measured.

### D. Data analysis

The mean distance between the first and second session measurements by the same observer was used to evaluate intraobserver agreement. Interobserver agreement was assessed comparing measurements between the observers for each landmark localization attempt.

Validation of the measurement method was performed with an independent group of subjects (n: 5) who had undergone both stereotactic MRI and CT imaging before GKR. CT scanning is not routinely used for radiosurgery planning in our center, but it is used selectively for targets near bone structures where target definition can be aided by CT imaging. This was the reason for the clinical stereotactic CT scan before GKR in these subjects (negative controls) and it provided an estimate of the overall variability of the evaluation method in terms of uncertainty of landmark localization between two different imaging techniques. The mean time interval between the beginning of the MRI scan and the CT scan in this group was 37.8 min (14‐73). As there were no treatment‐related stresses applied to the frame in this group, it was assumed that no frame displacement occurred in these patients.

The distance between two points in the Euclidian space was calculated using the equation below:

(1) $$d(A,B)=\sqrt{{\left(Ax-Bx\right)}^{2}+{\left(Ay-By\right)}^{2}+{\left(Az-Bz\right)}^{2}}$$

The mean distance between the location of the landmarks in the MRI and CT scan was calculated and reported for each patient to combine multiple observations of the landmarks. The individual localization attempts were used for evaluation of differences between the groups and logarithmic transformation of the distance data was done to enable comparison between the groups using independent sample's *t*‐test. Data analysis was performed using the Statistical Package for the Social Sciences (IBM SPSS version 22).

## III. RESULTS

**Figure 3:**
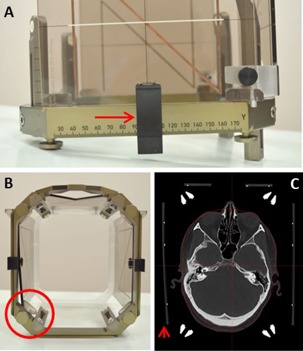
Fiducial indicator box accidentally misplaced at post‐GKR CT scan. Lateral view (a) of the stereotactic frame with the CT fiducial indicator box in an apparently normal position with the lateral clip adequately secured (red arrow). An inferior view (b) of the frame shows the right posterior locating pin of the indicator box displaced towards the midline (red circle) that resulted in an erroneous position of the fiducials in the CT scan ((c), arrowhead).

The mean frame‐on time defined as time difference between the beginning of the pre‐GKR MRI and post‐GKR CT scan in the study subjects was 157.8 min (89‐298). Two patients in the study group, one of them undergoing GKR for AVM, had the CT fiducial indicator box accidentally misplaced at post‐GKR CT scanning and this resulted in a considerable mismatch between the location of the landmarks in the MRI and CT scans in these two subjects (cases 13 and 17). As shown in Fig. 3, the right posterior locating pin of the fiducial indicator box was not seated in the corresponding hole in the frame ring but it was displaced towards the midline allowing the box to rest on the frame, and the lateral clips to be secured, in an apparently normal position. The center of the locating pin was misaligned by 7.5 mm from its normal position in both cases. This error was detected during definition of the CT scans in stereotactic space, which yielded mean and maximum fiducial errors of 1.6 and 4.6 mm, respectively. As expected, the discrepancy in the location of the landmarks between the scans was more evident on the right side and mainly affected the x‐axis. The mean distance between the location of the landmarks in the pretreatment MRI and post‐treatment CT in these two patients was 1.46 mm and the maximum difference noted was 2.24 mm. These two cases accidentally simulated the scenario of a frame displacement, and they were used as a positive control group for comparison. They were therefore excluded from the main study group, which was finally composed of 38 patients.

The fiducial error estimates of the definition of the T2‐weighted MRI and CT scans in stereotactic space in the main study group are shown in Table 2. As expected, the mean fiducial error of MRI scans (0.44 mm) was slightly larger than for CT scans (0.26 mm). Measurement of the position of the four landmarks was successfully accomplished in all but two patients. These were a subject with an AVM whose left cochlea was not included in the scan, and a patient with a left‐sided vestibular schwannoma who had undergone previous translabyrinthine excision of the tumor and the SSC was not visible.

**Table 2:** Fiducial error in stereotactic definition of MRI and CT scans for the main study group. The pretreatment MRI scan (T2‐weighted) and post‐treatment CT scan (bone window) were independently defined in stereotactic space using GammaPlan. Fiducial errors in the definition process for 38 patients included in the study group are summarized.

*Study Group (n:38)*	*Pre GKR MRI Scan T2‐weighted*	*Post‐GKR CT Scan Bone Window*
Mean error (mm)	0.44	0.26
(95%CI); SD	(0.42–0.46);0.06	(0.23–0.29);0.09
Maximum error (mm)	1.09	0.71
(95%CI); SD	(1.05–1.13);0.13	(0.63–0.79);0.24

The total number of coordinate measurements was 4272 as follows: 45 (subjects)×(landmarks)×2 (observers)×2 (repeated measurements)×2 (MRI and CT scans)×3 (x,y,z)−48 coordinate measurements for the two missing landmarks, as mentioned above. These measurements were obtained from three groups of patients — five subjects with both scans done before GKR and no plausible frame displacement (negative controls), two cases with misplaced indicator boxes at post‐GKR CT simulating a frame displacement (positive controls), and the main group of patients under investigation who underwent MRI scan before and CT scan after GKR (study group: 38). Table 3 shows the estimates of variability between repeated measurements by the same observer on the same scans (intraobserver variability) and between the observers (interobserver variability) in the three groups. Repeated measurements by the same observer on the same scans were consistent across the groups, with a mean distance between the first and second measurement of 0.25 mm. These estimates were similar for measurements obtained from CT (0.25 mm; 95% CI: 0.23–0.27) and MRI scans (0.24 mm; 95% CI: 0.22–0.26), p=0.3. The mean distance between measurements of the same landmark by different observers in the study group was higher at 0.59 mm (95% CI: 0.57–0.61).

The difference between the location of the landmarks in the MRI and CT scans for the three groups is shown in Table 4. The distance observed in the negative control group (0.63 mm; 95% CI: 0.57–0.70; SD: 0.29) represents the overall consistency of the evaluation method and provides an estimate of the minimum displacement that could possibly be detected by the study. The positive control group, with a mean distance between the location of the landmarks in the MRI and CT scans of 1.46 mm (maximum difference of 2.24 mm), demonstrates that the evaluation method is capable of detecting a discrepancy between the MRI and CT scans if there was one.

The mean distance between the location of the landmarks in the pre‐GKR MRI and post‐GKR CT scans for the study group was 0.71 mm (95% CI: 0.68–0.74; SD:0.32). The mean difference for each individual patient was below 1 mm. The estimates of difference between the MRI and CT coordinate measurements in the study group were consistent across the landmarks (i.e., cochleae: 0.71 mm (95% CI: 0.67–0.75) vs. 0.70 mm (95% CI: 0.67–0.74) for the SSC measurements, p=0.99).

**Table 3:** Intraobserver and interobserver variability of the measurements.

	*Intraobserver Variability Mean Distance in mm (95%CI) SD; max*	*Interobserver Variability Mean Distance in mm (95%CI) SD; max*
Negative controls	0.25 (0.22–0.29)	0.41 (0.34–0.47)
n: 5; obs: 80	0.16; 0.70	0.26; 1.14
Positive controls	0.21 (0.17–0.25)	0.57 (0.47–0.67)
n: 2; obs: 32	0.12; 0.45	0.26; 1.14
Study group	0.25 (0.23–0.26)	0.59 (0.57–0.61)
n:38; obs: 600	0.25 (0.23–0.26)	0.59 (0.57–0.61)

Negative controls=patients who underwent both MRI and CT scans before GKR; Positive controls=two patients with an accidentally misplaced fiducial indicator box at post‐GKR CT scanning (simulating a frame displacement); Study group=main group of subjects under investigation who underwent MRI scanning before and CT imaging after GKR (n:38); n=number of subjects; obs=number of observations.

**Table 4:** Distance (mm) between the location of the landmarks in the MRI and CT scans. The negative control group had both MRI and CT scans before GKR. The fiducial indicator box was accidentally misplaced at post‐GKR CT scanning in two cases (positive controls), simulating a frame displacement. The study group underwent MRI scanning before and CT imaging after GKR (n: 38). Subjects in the AVM group also had DSA for radiosurgery planning which involves longer frame‐on times and additional frame stress events.

*Group*	*Distance in Landmark Location MRI–CT Mean Distance in mm (95%CI) SD; max*
Negative controls	0.63 (0.57–0.70)
n:5; obs: 80	0.29; 1.36
Positive controls	1.46 (1.36–1.56)
n:2; obs: 32	0.28; 2.24
Study group	0.71(0.68–0.74)
n:38; obs: 600	0.32; 1.76
Study group excluding AVM	0.69 (0.66–0.72)
n: 30; obs: 476	0.31; 1.76
AVM only	0.78 (0.72–0.84)
n:8; obs: 124	0.34; 1.75

n=number of subjects; obs=number of observations.

**Figure 4:**
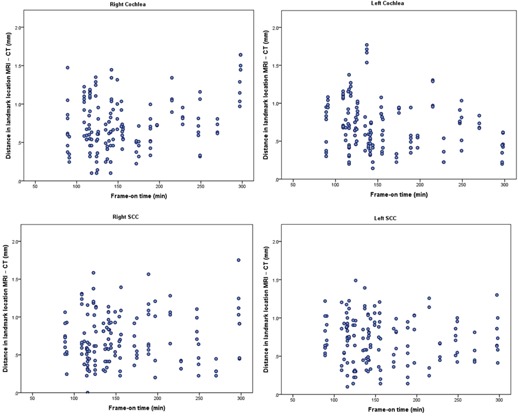
Difference between the MRI and CT coordinate measurements vs. frame‐on time in the study group for each of the landmarks analyzed. No correlation is seen between the frame‐on time and the estimated difference between the MRI and CT coordinate measurements for the cochlea and SCC bilaterally.

There was no correlation between frame‐on time and the difference between the MRI and CT coordinate measurements in the study group (Spearman's correlation coefficient: −0.03; p=0.4) and this is shown in Fig. 4 for each of the landmarks analyzed.

The group of patients with AVM, who also underwent DSA and so were subject to longer frame‐on times and additional frame stress events, showed slightly larger estimates of difference between the location of the landmarks compared to subjects with no additional imaging in the study group, 0.78 mm (95% CI: 0.72–0.84) vs. 0.69 mm (95% CI: 0.66–0.72), p=0.016. This is, however, a minimal difference (0.1 mm) and the mean difference in landmark location for all AVM patients remained submillimeter.

**Figure 5:**
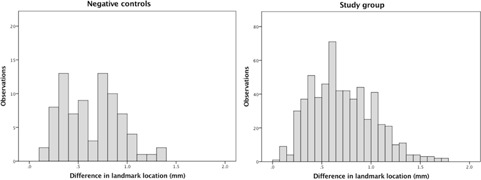
Distribution of the distance (mm) between the location of the landmarks in the MRI and CT scans for the negative controls and the main study group. Negative controls (left) underwent both MRI and CT scans before GKR. Study group (right) were main group of subjects under investigation who underwent pretreatment MRI scanning and CT imaging after GKR.

As shown in Fig. 5, the distribution of the distance between the location of the landmarks in the MRI and CT scans for the negative control and the study groups are similar and no statistical difference was noted between these groups (p=0.06). The maximum difference observed between the location of the landmarks in the MRI and CT scans for the negative control group was 1.36 mm. Outlier values in the study group above 1.5 mm were considered beyond the maximum uncertainty justifiable by the slice thickness and underwent further review.

**Figure 6:**
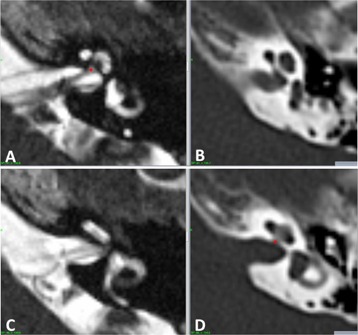
Discrepancy between MRI and CT imaging planes causing overestimation of the difference seen in the outliers (case 33): (a) the red cross shows an acceptable coordinate measure of the location of the cochlea in the pre‐GKR MRI scan (x, y, z: 127.1, 99.5, 135.9). The next MRI slice does not show the cochlear aperture (c). The images in the right show the cochlea bisected at two levels by the CT scan, one closer to the top (d) and the second closer to the bottom of the cochlea (b). The CT measurements for the study (x, y, z: 127.6, 99.5, 134.2) were taken from the superior slice (d) overestimating the difference in the z‐axis by the same magnitude as the slice thickness.

Ten pairs of MRI‐CT measurements in the study group, out of a total of 600, differed by more than 1.5 mm. Four of these outliers were observed in the left cochlea of a single patient by both observers on both occasions (case 33). This patient presented the maximum difference noted in the study group (1.76 mm), but no similar difference was noted in the measurements of the ipsilateral SSC or the contralateral landmarks. Similarly, three outliers were observed in the right cochlea of a second subject by both observers, in two occasions by one of them, but again no large difference was noted in the ipsilateral SSC or the contralateral landmarks (case 20). A thorough review of these datasets demonstrated a discrepancy between the MRI and CT imaging planes which resulted in an overestimation of the difference in the z‐axis, comparable to the 1.5 mm slice thickness. As shown in Fig. 6, the CT slices bisected the cochlea at two levels, one closer to the top and the second closer to the bottom, rather than at the middle where the cochlear aperture would have been better visualized. This resulted in the landmark being measured at two different levels in the MRI and CT scans. The three remaining outliers were isolated inconsistencies measured by a single observer, in a single landmark, and in three different patients (cases 22, 23, and 46). These were also detected in only one of the two measurements made by the same observer and were considered genuine observer errors.

## IV. DISCUSSION

This study aimed to evaluate a commonly overlooked factor contributing to the global accuracy of GKR. It is the assumption that the stereotactic frame stays still during the treatment procedure and does not move. The location of four defined landmarks were measured in the planning MRI scan and compared to their observed position in a CT scan acquired immediately after treatment. If the stereotactic frame was completely stable and it was possible to measure the position of the landmarks in the two scans without any uncertainty, the difference between the first and second observations would have been zero. This was not the outcome of the study. A quantifiable difference of 0.71 mm (95% CI: 0.68–0.74; SD: 0.32) between the position of the landmarks in the pre‐GKR MRI and the post‐GKR CT scan was observed. This difference reflects the effect of three elements of the evaluation process — the variability of the measurements within and between the observers, the uncertainty of the imaging techniques used for the study, and a potential degree of frame displacement. These factors were quantified in an independent group of subjects (negative controls) who underwent both scans before GKR with minimal time and frame stress events between them. The mean difference between the location of the landmarks in the MRI and CT scans in this group was 0.63 mm (95% CI: 0.57–0.70; SD: 0.29). This is considered to be the best estimate of values with no frame displacement under the research conditions and provides a threshold of minimum displacement that could have possibly been detected by our study.

The difference observed in the study group (0.71 mm; 95% CI: 0.68–0.74; SD: 0.32) was not statistically different to the overall uncertainty of the evaluation method as observed in the negative control group (0.63 mm; 95% CI: 0.57–0.70; SD: 0.29). It is not possible to assert that frame displacement was zero because the difference found in the study group is equivalent to the uncertainty of the measurement method. It is also not possible to estimate from the study how much of the submillimeter difference observed in the study group is potentially explained by frame displacement. It can, however, be confidently concluded that no systematic frame displacement larger than 0.71 mm occurred in the study group.

### A. Intra‐ and Interobserver variability

The variability of the measurements in our study is one of the factors contributing to the overall uncertainty of the results. The mean distance between repeated measurements by the same observer was 0.25 mm and as expected a larger mean distance was noted between measurements by different observers (0.59 mm). These estimates reflect not only the individual skills of the observers, but also the size and nature of the landmarks, which should be sensitive to potential frame displacement but also reliably identifiable in the scans. The cochlear apertures and the summit of the anterior post of the SSC were thought to be good landmarks for the study because they are nonmidline structures sensitive to potential rotational displacement of the frame, they are clearly visible in both CT and T2‐weighted MRI scans, and small enough in size for their location to be acceptably summarized by a single set of X, Y and Z coordinates. There is, however, a degree of imprecision in using a single point to define the location of a three‐dimensional structure.

The uncertainty of the coordinate measurements also derives from the fact that a landmark could be bisected by the scan slices at two different levels. The resultant discrepancy reflects the arbitrary quantization of the imaging planes rather than the exact position of the landmarks in the stereotactic system. The location of the SSC was measured in the coronal plane to minimize this source of error, and also a consensus was reached between the observers in two cases where one of the cochlear apertures was bisected by two different slices. This, however, applied only to measurements obtained from the same scan and it was not possible to adjust for slice misalignment when comparing the position of the cochlea (as measured in the axial plane) between two different scans. This error was particularly obvious in two of our study group patients where one of the cochleae was bisected at two levels by the post‐GKR CT scans (Fig. 6). This resulted in coordinate values being obtained from different anatomical structures due to suboptimal visualization of the landmark in the scans. The distance noted between these outlier observations was between 1.5 and 1.76 mm and this clearly reflects the slice thickness. No such difference was noted in the ipsilateral SSC of the same patients, which was measured in the coronal plane or the landmarks in the contralateral side. This reinforces the evidence that the outliers in our study are caused by the arbitrary slice misalignment between the scans rather than a displacement of the stereotactic frame. This would have been reduced if thinner slices were used for the imaging procedure. The coronal MRI and CT planes, reformatted from the axial images, have an effective slice thickness of 0.47 mm (voxel dimensions: 0.47×0.47×1.5 mm) and this has a lesser effect for localization of the SSC in the coronal plane. The cochleae, however, were not considered less suitable landmarks for comparison, and their difference in apparent location between the MRI and CT scans was found to be comparable to the same estimates with the SCC (cochleae: 0.71 mm, 95% CI: 0.67–0.75; SCC: 0.70 mm, 95% CI: 0.67–0.74, p=0.99).

### B. Localization uncertainty due to imaging techniques

The validity of the conclusions reached by this study rest on the assumption that it is possible to reliably identify the same landmarks in two different imaging techniques: MRI and CT. Figure 2 demonstrates the high‐quality visualization of the landmarks in both images. Nonetheless, technical factors inherent to the imaging technique will introduce some uncertainty to the localization coordinates. MRI distortion is the most significant of these factors and was recently characterized by Nakasawa et al.^(9)^ They demonstrated that the maximum absolute error of coordinates in each dimension using 1.5 T MRI and the Leksell Frame G with titanium fixation screws is between 1 and 2 mm. This is considerably higher than the estimate of distortion of the MRI unit used in our study, which was found to be a mean of 0.46 mm and a maximum of 0.88 mm using a GRID3D known target phantom (Modus Medical Devices Inc. London, Canada). For our CT scanner, a mean of 0.41 mm and a maximum of 0.70 mm were measured with the same phantom. Combining the errors for these two modalities in quadrature yields mean and maximum errors of 0.62 mm and 1.12 mm, respectively. This mean error is remarkably close to the mean difference in the negative controls group, adding weight to our assumption that the negative controls group is a valid estimate of our uncertainties.

**Figure 7:**
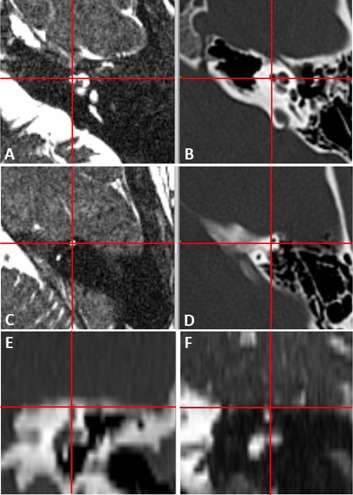
Submillimeter discrepancies between the location of the landmarks in the MRI and CT scans attributed to imaging distortion. The red lines cross in the center of the cochlear canal at the most medial spiral turn in the pre‐GKR MRI scan (a) but slightly off‐center in the post‐GKR CT scan (b). Similar discrepancies between 0.5 and 1 mm were seen in the axial view ((c) and (d)) and coronal view ((e) and (f)) of the SCC (case 16).

The uncertainty caused by imaging distortion contributes to the apparent differences in landmark positions seen between the MRI and CT scans in some cases where the same stereotactic point seems to show a slightly different anatomical position in the scans (Fig. 7). This phenomenon has been described by Karlsson et al.^(10)^ who reported a mismatch above 1.5 mm between two stereotactic MRI scans of the same subject and concluded it was the result of MRI distortion artifact. Pollock et al.^(11)^ has also reported the importance of MRI distortion in stereotactic radiosurgery planning and suggested that imaging distortion could have partly caused some of their recurrent vestibular schwannomas to receive less than the prescribed dose to the entire tumor volume. This is also relevant to our outlier measurements observed in the cochlea, where CT imaging is likely to better characterize the anatomy of the bony structure compared to MRI.

Further testing of the measurement method was possible in our study due to unintentional misplacement of the CT indicator box in two cases (positive controls). The simulated displacement was evidenced in the coordinate measurements as a mean difference between the position of the landmarks in the MRI and CT scans of 1.46 mm (95% CI: 1.36–1.56). The maximum discrepancy observed was 2.24 mm, which is considerably larger than the maximum observed difference in the study group. This error was effectively detected by GammaPlan (Elekta AB) and the planner was made aware of this through unusually high fiducial errors during the stereotactic definition of the CT scans. This would not have been the case if a real frame shift occurred because there is currently no mechanism in place to detect such a displacement throughout the treatment procedure. The development of the Leksell Gamma Knife and its integration with movement tracking and live image guidance systems in the recently launched Gamma Knife Icon is expected to address potential geometric inaccuracies throughout the treatment procedure.^(12)^

The scenario of a misplaced indicator box is clearly geometrically simpler than a multiplane or rotational frame displacement, but it can provide some understanding of the degree of error that would be added to a treatment if the stereotactic frame displaced. In the two cases with a misplaced indicator box in our study, the right posterior locating pin of the fiducial indicator box was displaced 7.5 mm towards the midline and the maximum error of the scan definition process was 4.6 mm. However, the maximum discrepancy observed between the location of the landmarks in the MRI and CT scans was only 2.5 mm, considerably lower than the potential error in the system. This reduction of error is caused by a redistribution of the inaccuracies across the scans when they are defined in stereotactic space.^(13)^ Similar benign error redistribution effects could occur if the frame displaced under specific conditions such as axial rotation with a centrally located target and this study did not specifically address the issue of potential rotational displacement of the frame. Further understanding of the effect of random geometrical uncertainties in the treatment procedure is needed to fully comprehend the clinical effect of potential frame displacement, and also for evaluation and development of frameless radiosurgery systems.^14^, ^15^

### C. Frame stability

Karlsson et al.^(10)^ evaluated the stability of the Leksell frame in a group of 18 patients who underwent high‐definition MRI scans before and after GKR. The reported mean distance between the average of repeated readings of the location of defined landmarks before and after GKR was 0.47 mm and it was concluded that the position of the stereotactic frame is stable throughout the treatment procedure. The difference reported by Karlsson and colleagues is considerably lower than that observed in our study and this is explained by the methodological differences. The comparison between the pre‐ and post‐GKR scans in their study was done using the calculated mean of repeated observations (mean position of localization), rather than each individual localization attempt, and this could result in erroneously favorable outcomes.^(2)^ Also, we used a different imaging technique before and after GKR, and the imaging slices were also thicker in our study (1.5 vs. 0.7 mm), which explains the increased uncertainty of our measurements.

The application accuracy of four commonly used stereotactic devices, including the Leksell frame, was challenged by Maciunas et al.^(2)^ in an attempt to evaluate the total accuracy conveyed by the entire visualization, calculation, and surgical systems working together. The study suggested a significant degree of error in the application accuracy of all the stereotactic instruments tested, but failed to specifically address the question of stability of the frame itself. Their experiment compared the true location of a target in a phantom with the position reached by a needle tip if the target coordinates were set in the stereotactic arc system. This comparison was performed several times using different CT slice thickness and the mean error was found to be within 1–2 mm when 1 mm slices were used and within 2–3 mm with 4 mm slices. These estimates of inaccuracy are considerably larger than the observed mean difference in our study and only our outliers are somewhere near the best results by this group. This is probably due to the fact that the findings of the Maciunas study relied on the poor geometric accuracy of earlier generation CT scanners, which are now known to yield errors of a few mm.^(16)^ The experiment designed by Maciunas and colleagues cannot be used as evidence against the stability or accuracy of the Leksell Frame G; however, it provides awareness of how slice thickness can influence the overall accuracy of stereotactic systems.

### D. Effect of frame weight bearing on the stereotactic frame

The paper by Maciunas et al.^(2)^ also claimed to evaluate the effect of frame weight‐bearing on the accuracy of the stereotactic systems and concluded that a mean displacement of 1.62 mm occurs if a weighting of 25 Kg is added to the Leksell Frame G. This is a significant weight unlikely to represent any clinical scenario and it is not possible to determine from the study report how the Leksell frame was fixed to the phantom's acrylic plastic base. Their results are more likely to reflect a technical fault on the specific experimental design rather than any clinically relevant instability of the frame. Nevertheless, the issue of frame weight‐bearing has not been fully addressed in the literature and our study provided the opportunity to compare a group of AVM patients, with additional frame stress events incurred in the process of DSA, and a group of subjects who were not exposed to these factors. The results demonstrated that the difference between the location of the landmarks in the pre‐ and post‐treatment scans is slightly higher in the AV M patients, who were subject to longer frame‐on times and additional frame stress events, compared to other subjects in the study group who underwent no additional imaging (0.78 mm vs. 0.69 mm; p=0.016). The difference between these two groups, although very small in magnitude (0.1 mm), is statistically significant, and it may be beneficial to reduce the number of frame stress events during the treatment procedure. This may also have implications for patients who are treated using trunnion fixation on Gamma Knife model B and C where patient frames are repeatedly stressed during docking for each isocenter.

## V. CONCLUSIONS

A comprehensive study design involving repeated measurements of the landmarks in 40 patients by two observers, along with validation of the evaluation method in an independent negative control group with no plausible frame displacement, demonstrated that the observed difference in the study subjects is equivalent to the overall uncertainty of the evaluation method. This provides reliable and realistic evidence of submillimeter stability of the stereotactic frame throughout the treatment procedure.

## ACKNOWLEDGMENTS

This study was funded by Queen Square Radiosurgery Centre

## COPYRIGHT

This work is licensed under a Creative Commons Attribution 4.0 International License.
